# Engineering hypertrophic cartilage grafts from lipoaspirate for critical‐sized calvarial bone defect reconstruction: An adipose tissue‐based developmental engineering approach

**DOI:** 10.1002/btm2.10312

**Published:** 2022-03-24

**Authors:** Ru‐Lin Huang, Rao Fu, Yuxin Yan, Chuanqi Liu, Jing Yang, Yun Xie, Qingfeng Li

**Affiliations:** ^1^ Department of Plastic and Reconstructive Surgery Shanghai Ninth People's Hospital, Shanghai Jiao Tong University School of Medicine Shanghai China; ^2^ Department of Plastic and Burn Surgery West China Hospital, Sichuan University Chengdu China

**Keywords:** adipose tissue, bone defect reconstruction, bone regeneration, developmental engineering, endochondral ossification, hypertrophic cartilage

## Abstract

Developmental engineering of living implants from different cell sources capable of stimulating bone regeneration by recapitulating endochondral ossification (ECO) is a promising strategy for large bone defect reconstruction. However, the clinical translation of these cell‐based approaches is hampered by complex manufacturing procedures, poor cell differentiation potential, and limited predictive in vivo performance. We developed an adipose tissue‐based developmental engineering approach to overcome these hurdles using hypertrophic cartilaginous (HyC) constructs engineered from lipoaspirate to repair large bone defects. The engineered HyC constructs were implanted into 4‐mm calvarial defects in nude rats and compared with decellularized bone matrix (DBM) grafts. The DBM grafts induced neo‐bone formation via the recruitment of host cells, while the HyC pellets supported bone regeneration via ECO, as evidenced by the presence of remaining cartilage analog and human NuMA‐positive cells within the newly formed bone. However, the HyC pellets clearly showed superior regenerative capacity compared with that of the DBM grafts, yielding more new bone formation, higher blood vessel density, and better integration with adjacent native bone. We speculate that this effect arises from vascular endothelial growth factor and bone morphogenetic protein‐2 secretion and mineral deposition in the HyC pellets before implantation, promoting increased vascularization and bone formation upon implantation. The results of this study demonstrate that adipose‐derived HyC constructs can effectively heal large bone defects and present a translatable therapeutic option for bone defect repair.

## 1 INTRODUCTION

The body's repair mechanisms are often unable to spontaneously heal large bone defects resulting from traumas, tumors, infections, and congenital malformations. These defects represent a significant medical concern and socioeconomic burden. Bone is the second most frequently transplanted tissue after blood, with over 2.2 million bone graft procedures performed annually worldwide, representing an economic burden of US $3 billion.^1^ Among all clinically available grafts, autologous bone is still considered the gold standard since it combines all necessary properties required for bone regeneration, namely, osteoconduction, osteoinduction, and osteogenesis.^2^ However, the current autologous bone grafting procedure can only be used to treat a small number of patients, mainly due to donor site morbidity, limited donor availability, and the high cost and complication rate.^3^, ^4^ Several alternatives to autologous bone grafting have emerged, such as allografts, xenografts, and synthetic grafts. These alternatives are available in various forms and in large quantities, but each has specific indications and limitations, and mixed results have been obtained.^5^, ^6^ Therefore, there is an urgent need for the development of a new generation of bone substitutes.

Tissue‐engineered bone substitutes consisting of cells, biomaterials, and bioactive molecules have become a viable approach for bone defect reconstruction.^7^, ^8^, ^9^ The traditional bone tissue engineering (BTE) approach mimics the process of an intramembranous ossification (IMO) pathway, by which mesenchymal stem cells (MSCs) are induced to undergo osteogenic differentiation and subsequently form a bone‐like matrix.^10^ A major drawback of such strategies is the limited size of the engineered constructs. In vitro osteogenic induction results in extensive matrix deposition on the surface of the construct, which hampers nutrient delivery and makes it difficult to scale up the size.^11^ Furthermore, extensive bone matrix on the surface hinders the invasion of blood vessels upon construct implantation. Thus, such strategies often fail due to avascular necrosis and core degradation resulting from poor perfusion.^12^, ^13^ Consequently, attention has shifted toward an alternative route of “developmental engineering,” which strives to stimulate in vivo developmental processes and initiate natural factors that govern cell differentiation and matrix production.^14^, ^15^ In contrast to IMO‐based BTE approaches, developmental engineering‐based strategies involve engineering cartilaginous constructs by replicating certain aspects of endochondral ossification (ECO).^16^, ^17^, ^18^ Briefly, MSCs are induced to differentiate into chondrocytes in vitro to form a hypertrophic cartilage (HyC) construct containing essential “biological instructions” that initiate the ECO process after implantation, and the defect is subsequently repaired via endochondral bone regeneration (EBR).^19^, ^20^, ^21^ This strategy offers a solution to the problems associated with limited size and poor vascularization after implantation. Chondrocytes within the cartilage intermediate can intrinsically resist hypoxic environments^22^ and induce neovascularization and ossification through the release of bioactive factors, including vascular endothelial growth factor (VEGF), bone morphogenic proteins (BMPs), and hydroxyapatite‐containing vesicles.^20^, ^23^ Furthermore, engineered HyC grafts have been reported to promote faster host integration and bone formation after implantation in vivo.^24^, ^25^, ^26^

However, clinical translation of developmental engineering‐based bone regeneration is still in the early stages. One of the main challenges is the complexity and variability of current EBR approaches. Various types of cells, including adipose‐derived stem cells (ASCs),^24^, ^27^ bone marrow‐derived stem cells (BMSCs),^9^, ^20^ induced pluripotent stem cells,^28^, ^29^ and periosteum‐derived cells (PDCs),^30^, ^31^ have been used for bone defect reconstruction via EBR. However, these cell‐based strategies frequently rely on a series of in vitro cell manipulation techniques, including cell isolation, in vitro expansion, and seeding onto scaffolds, which not only hamper clinical transplantation but also impair the in vivo performance of the engineered implants.^32^, ^33^, ^34^ We previously used fractioned human subcutaneous adipose tissue (nanofat^35^) rather than ASC‐seeded bioscaffolds for endochondral bone engineering. The resulting constructs developed a HyC phenotype and demonstrated better endochondral bone formation capacities than ASC‐seeded collagen sponges in ectopic bone formation models.^36^ In this approach, adipose tissue not only serves as a stem cell niche for tissue regeneration but also provides an innate extracellular matrix (ECM) that acts as a native scaffold and supports stem cell proliferation and differentiation during matrix synthesis and remodeling. These proof‐of‐concept studies provide a clinically translatable alternative to bone defect repair; however, preclinical studies in animal models are required to evaluate the feasibility of this approach for repairing bone defects.

In this study, we propose an adipose tissue‐based developmental engineering strategy for reconstruction of critical‐sized bone defects. To do so, we engineered HyC constructs from human lipoaspirate by sequential in vitro proliferative culture, chondrogenic differentiation, and hypertrophic induction. The resulting HyC pellets were subsequently implanted into critical‐sized calvarial defects in a nude rat model and compared with a bone substitute decellularized and demineralized bone matrix (DBM) graft to evaluate their ability to promote EBR and remodeling in orthotopic sites.

## 2 METHODS

### 2.1 Generation of *Adiscaf* constructs from human adipose tissue

Human adipose tissue samples were collected from patients who underwent liposuction at the Department of Plastic and Reconstructive Surgery, Shanghai Ninth People's Hospital, Shanghai Jiao Tong University School of Medicine (*n* = 8; average age, 30.6 ± 9.5 years), and written consent was obtained preoperatively. The study protocol was approved by the ethics committee of our institute (ethics number: SH9H‐2021‐A974‐SB). Human *Adiscaf* constructs were generated as previously described.^36^, ^37^ Briefly, lipoaspirate was washed, minced, and loaded into a 20‐ml syringe that was connected to another syringe through a three‐way stopcock. Then, the lipoaspirate was fractionated by shifting the content from one syringe to another 30 times. The emulsified adipose tissue, namely, nanofat,^35^ was seeded into six‐well agarose‐coated plates. The nanofat was cultured in proliferative medium for 3 weeks, consisting of alpha‐minimal essential medium supplemented with 10% fetal bovine serum, 1% HEPES, 1% sodium pyruvate, 1% penicillin–streptomycin glutamine (all from Gibco), 10^−5^ M ascorbic acid, 10^−7^ M dexamethasone (both from Sigma‐Aldrich), 5 ng/ml fibroblast growth factor‐2, and 10 ng/ml platelet‐derived growth factor (both from R&D Systems). After 3 weeks, 4‐mm biopsy punches were taken from the *Adiscaf* constructs and placed into 12‐well agarose‐coated plates for an additional week of proliferative culture.

### 2.2 Cell isolation, expansion, and differentiation induction

Stromal vascular fraction (SVF) cells harvested from lipoaspirate and ASCs from *Adiscaf* constructs were used as reference for the efficiency of differentiation capacity. SVF cells were isolated after enzymatic digestion of adipose tissue and centrifugation as previously described.^37^ In vitro osteogenic, adipogenic, and chondrogenic differentiation were induced as previously described.^37^ Briefly, 5 × 10^5^ cells were seeded onto Ultrafoam (4 mm in diameter, 1‐mm thick; Davol). The constructs were then cultured in chondrogenic medium, composed of serum‐free CM supplemented with 10^−7^ M dexamethasone, 0.01 mM ascorbic acid, 10 ng/ml transforming growth factor‐β_3_ (TGF‐β_3_), and 10 ng/ml bone morphogenetic protein 6 (BMP‐6; both from R&D Systems) for 4 weeks.

### 2.3 Chondrogenic differentiation and hypertrophic induction of *Adiscaf* pellets

After 4 weeks of proliferative culture, *Adiscaf* pellets were submitted for 4 weeks of chondrogenic differentiation followed by 2 weeks of hypertrophic induction. For chondrogenic differentiation, the *Adiscaf* pellets were cultured in chondrogenic medium consisting of serum‐free medium supplemented with 10 ng/ml TGF‐β_3_, 10 ng/ml BMP‐6, 10^−5^ M ascorbic acid, and 10^−7^ M dexamethasone. For hypertrophic induction, the *Adiscaf* pellets were cultured in hypertrophic medium consisting of serum‐free medium supplemented with 0.01 M β‐glycerophosphate (Sigma‐Aldrich), 10^−7^ M dexamethasone, and 10^−5^ M ascorbic acid.

### 2.4 Flow cytometry analysis

Prior to acquisition, cells (3–5 × 10^5^ cells) were suspended in 200 μl of 0.5% bovine serum albumin in phosphate‐buffered saline (PBS) containing conjugated antibodies against the indicated protein or an isotype control and were incubated for 30 min at room temperature. The following antibodies were used: CD45‐FITC (555482), CD90‐APC (559869), CD73‐PE (561014), CD34‐APC (555824), CD146‐PE (561013), and CD31‐PE (560983, all from Becton Dickinson, Franklin Lakes, and used at a dilution of 1:50). Cells were washed twice with FACS buffer, suspended in PBS, and analyzed with a FACS‐Calibur flow cytometer (Becton Dickinson).

### 2.5 Glycosaminoglycan and DNA quantification

Samples were digested with proteinase K for 16 h at 56°C. For glycosaminoglycan (GAG) quantification, digested *Adiscaf* constructs were incubated in 1 ml of dimethylmethylene blue (DMMB) solution on a shaker at room temperature for 30 min. Precipitated DMMB–GAG complexes were centrifuged, and supernatants were discarded. Complexes were dissolved in decomplexion solution at 60°C, absorption was measured at 656 nm, and GAG concentrations were calculated using a standard curve prepared with purified bovine chondroitin sulfate. DNA content was measured with the DNeasy Blood & Tissue Kit (Qiagen) according to the manufacturer's instructions.

### 2.6 Scanning electron microscopy

Samples were fixed with 0.25% glutaraldehyde at 4°C overnight and then washed with PBS. The samples were subsequently dehydrated with alcohol, coated with gold, and imaged with an FEI XL‐30 SEM microscope (FEI).

### 2.7 Animal experiments and surgical procedures

For orthotopic implantation, twenty 6–8‐week‐old male nude rats (Charles) were used to establish the critical‐sized calvarial defect model. The rats were anesthetized by isoflurane, the scalp area was shaved, and a midline incision was made to expose the cranium. Two 4‐mm diameter defects were drilled by a trephine bur with normal saline irrigation. The bone defects were left untreated (blank group, *n* = 20), implanted with HyC pellets (HyC group, *n* = 10) or implanted with DBM (DBM group, *n* = 10; Shanghai Exceller Biomedical Company) (Figure 4a,b). The periosteum, subcutaneous tissue, and skin were sutured layer‐by‐layer after implantation. The rats had free access to food and water thereafter. The animals were sacrificed 6 or 12 weeks postimplantation, and the calvarial bones were harvested and examined. For ectopic implantation, DBM grafts were subcutaneously implanted into 4–6‐week‐old male Balb/c nude mice (Charles), as previously described.^38^, ^39^

### 2.8 Microcomputed tomography

Microcomputed tomography (microCT) data were acquired from the rats immediately and at 6 and 12 weeks postimplantation using high‐resolution microCT (Skyscan1176) at 50 kV and 400 μA. Transmission images (360°) were acquired with an incremental step size of 0.25°. Volumes were reconstructed through CTVox v.3.0 at a voxel size of 8 μm. Thresholding, segmentation, and 3D measurements were performed using CTAn (Skyscan) software.

### 2.9 Histological and immunohistochemical staining

Samples were fixed in 4% paraformaldehyde overnight, decalcified using EDTA (if necessary), embedded in paraffin and sliced at a thickness of 5 μm. The sections were stained with hematoxylin and eosin (H&E; Sigma‐Aldrich), Safranin‐O, Masson trichome, Alizarin red (both from Solarbio), or Movat's pentachrome staining (Abcam). Sections were also analyzed for tartrate‐resistant acid phosphatase (TRAP) activity using the leukocyte acid phosphatase kit (Sigma‐Aldrich).

For immunohistochemical staining after rehydration, the sections were blocked with 5% goat serum and incubated with the following primary antibodies: rabbit anti‐human collagen type I (Col I; ab138492), rabbit anti‐human collagen type II (Col II; ab34712), mouse anti‐human collagen type X (Col X; ab49945), rabbit anti‐human VEGF (ab46154), rabbit anti‐human bone sialoprotein (BSP; ab52128), rabbit anti‐human matrix metalloproteinase‐9 (MMP‐9; ab38898), rabbit anti‐human MMP‐13 (ab39021), rabbit anti‐human BMP‐2 (ab6285), rabbit anti‐human nuclear mitotic apparatus protein (NuMA; ab241470), and rabbit anti‐human and rat Osterix (ab209484; all from Abcam). After sequential incubation with a biotinylated secondary antibody and an ABC‐alkaline phosphatase complex, specific staining was revealed by using Fast Red (Dako).

### 2.10 Histomorphometric quantification

Quantitative analysis of new bone area was performed on representative H&E staining images by ImageJ software (National Institutes of Health). Quantitative analysis of blood vessels was performed on representative Movat's staining images by ImageJ, in which the blood vessels were stained red with luminal structure. Four different fields of view were randomly selected in four specimens within the defect area under ×20 magnification (*n* = 16). The vessel density was named blood vessels/mm^2^ by calculating the number of blood vessels per area of each image. Quantitative analysis of NuMA‐positive cells was performed by ImageJ and expressed as NuMA^+^ cells (%). Four different fields of view were randomly selected in four specimens of the HyC group under ×20 magnification (*n* = 16).

### 2.11 Statistical analysis

All experiments were performed at least in triplicate per condition. Data were presented as the mean ± standard deviation. Data were compared with one‐way analysis of variance with Tukey's multiple comparison test or Student's *t*‐tests to determine significant differences between two groups. The results were considered significantly different when *p* values were lower than 0.05. Statistical analysis was performed with GraphPad Prism 9.0 software (GraphPad Software).

## 3 RESULTS

### 3.1 In vitro generation of *Adiscaf* constructs from human adipose tissue

**FIGURE 1:**
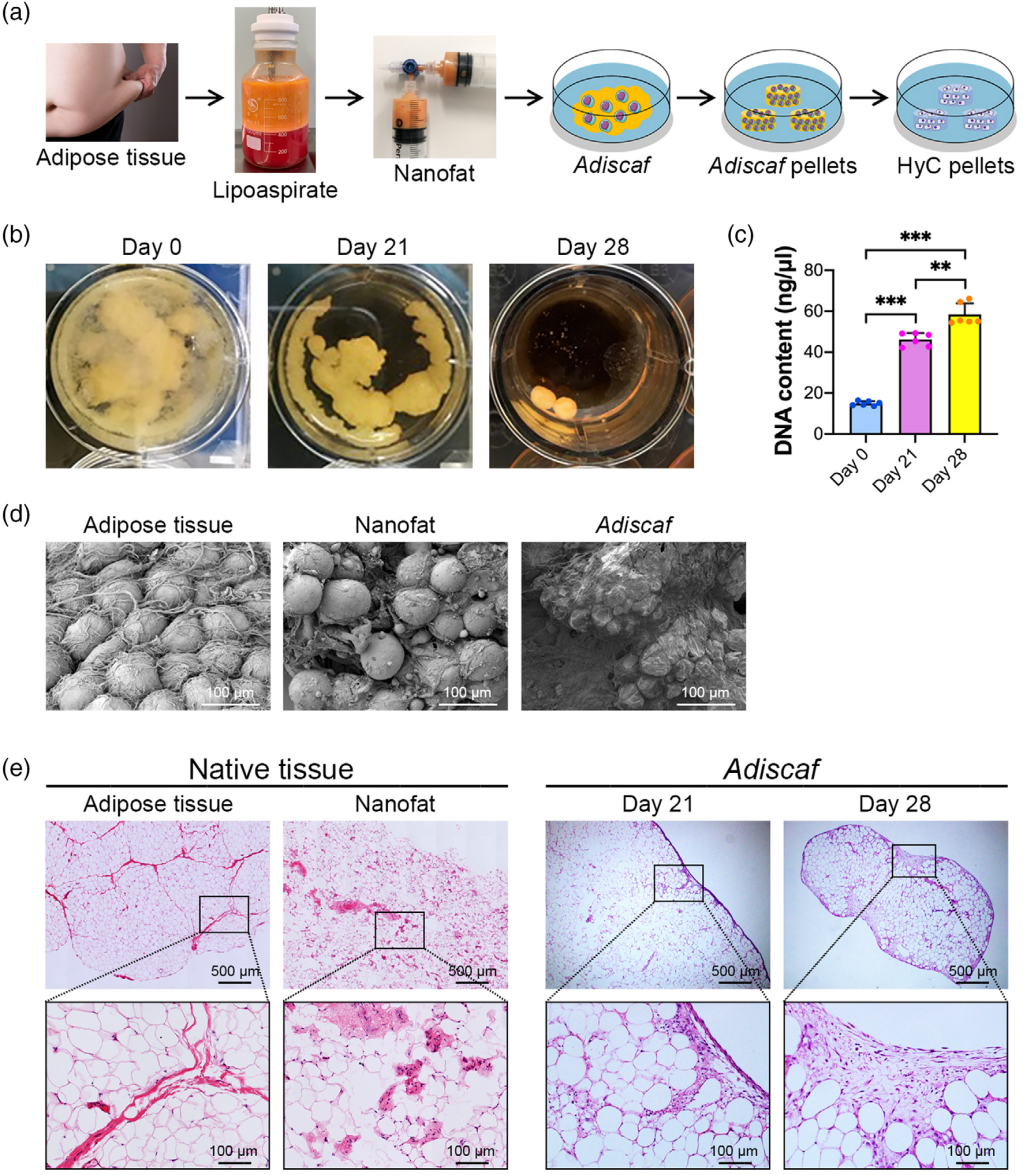
Characterization of the *Adiscaf* constructs. (a) Schematic overview of the process of bioengineering HyC pellets from human adipose tissue in vitro. (b) In vitro culture of nanofat tissue in proliferative medium for 21 days as condensed adipose tissue and an additional 7 days as tissue pellets. (c) Quantification of the DNA contents at Day 0, Day 21, and Day 28 (*n* = 6; **p < 0.01, ***p < 0.001). (d) SEM images of native adipose tissue, nanofat tissue, and *Adiscaf* constructs. (e) H&E staining of native adipose tissue, nanofat, and *Adiscaf* constructs and pellets. H&E, hematoxylin and eosin; SEM, scanning electron microscopy

Human adipose tissue was collected from patients undergoing liposuction and utilized for HyC pellet generation, as shown in Figure 1a. After 21 days of proliferative culture, the loose nanofat tissue (Figure 1b, left) aggregated and condensed, consistent with previous descriptions of *Adiscaf* constructs^36^, ^37^ (Figure 1b, middle). Then, 4 mm *Adiscaf* pellets were punched from the constructs and exposed to proliferative culture conditions for an additional week to generate smaller, denser *Adiscaf* pellets (Figure 1b, right). Quantification of the DNA content of nanofat (Day 0), *Adiscaf* constructs (Day 21), and *Adiscaf* pellets (Day 28) confirmed that the cell number was increased three‐fold during the generation of *Adiscaf* constructs and was increased four‐fold in the *Adiscaf* pellets (Figure 1c). Scanning electron microscopy (SEM) images demonstrated the surface morphology of the native adipose tissue, nanofat, and *Adiscaf* constructs. The native adipose tissue demonstrated a cluster of adipocytes supported by a delicate network of reticular fibers (Figure 1d, left). However, the nanofat tissue structure was smaller and looser, and many damaged adipocytes and fibers were present (Figure 1d, middle). After 3 weeks of in vitro proliferation, the surface of adipocytes was covered with a newly formed ECM (Figure 1D, right). H&E staining showed that the native adipose tissue had large intact adipocytes residing in small groups within loose irregulate connective tissues. Intersyringe shuffling disrupted this structure and resulted in a large number of damaged adipocytes and fibers (Figure 1e, left). After 3 weeks of culture in proliferative medium, the loose nanofat reaggregated and formed relatively dense adipose tissue with abundant proliferating cells and ECM present among the adipocytes, especially at the periphery of the *Adiscaf* constructs, consistent with the surface morphology results observed under SEM. An additional 1 week of culture further enhanced cell proliferation and ECM deposition within the *Adiscaf* constructs (Figure 1e, right).

### 3.2 *Adiscaf* constructs possess greater potency than SVF‐seeded Ultrafoam constructs on in vitro HyC engineering and in vivo bone formation

**FIGURE 2:**
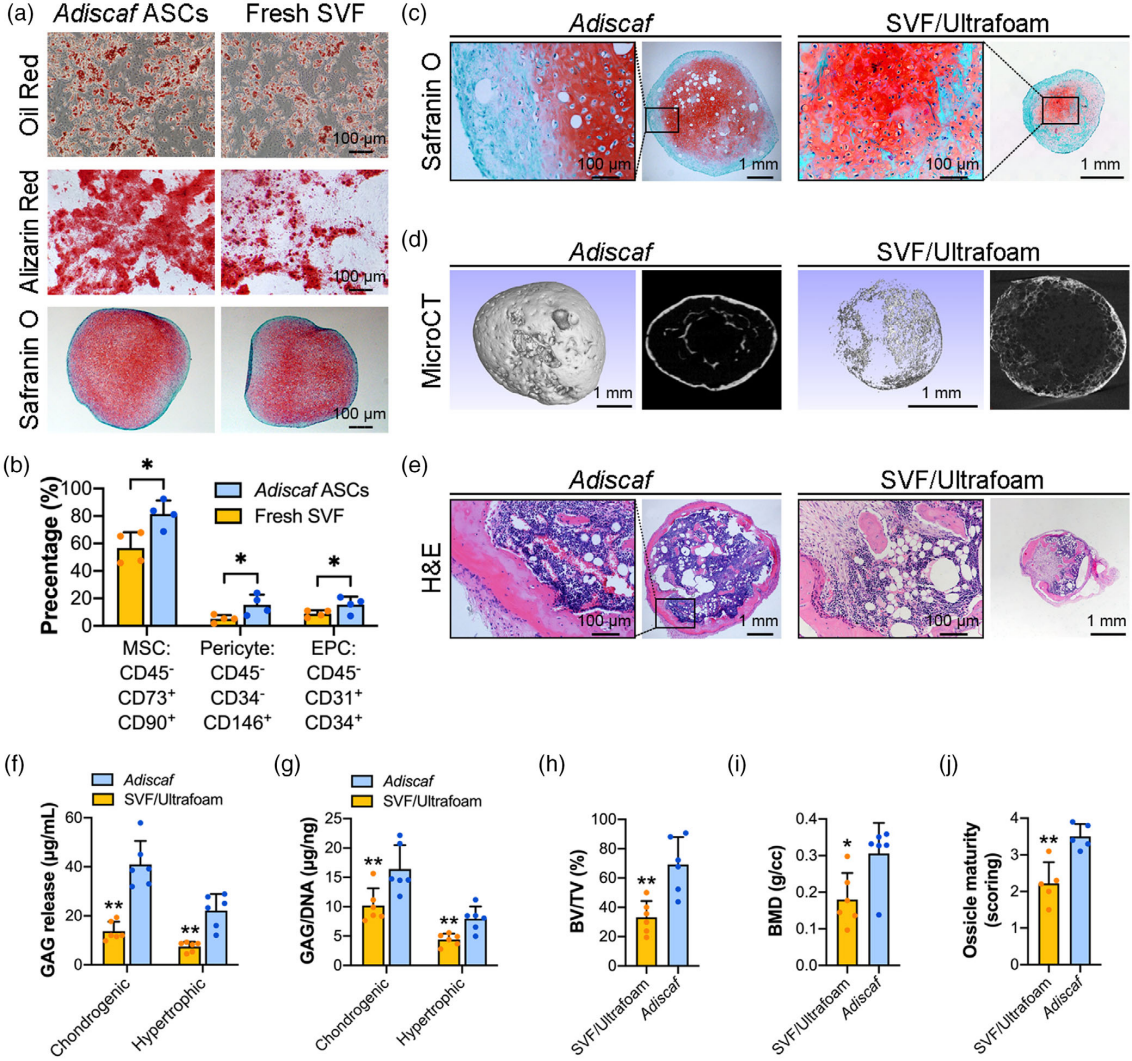
*Adiscaf* constructs showed higher efficiency than SVF/Ultrafoam constructs in hypertrophic graft engineering and ectopic bone regeneration. (a) ASCs derived from *Adiscaf* constructs and fresh SVF cells derived from lipoaspirates were induced to differentiate into adipocytes (*above*), osteoblasts (*middle*), and chondrocytes (*bottom*). (b) Cell phenotype analysis of the *Adiscaf*‐derived ASCs and fresh SVF by flow cytometry. Quantification data are presented as the percentages of living cells among different subpopulations of cells: MSCs (CD45^−^, CD73^+^, CD90^+^), pericytes (CD45^−^, CD34^−^, CD146^+^), and EPCs (CD45^−^, CD31^+^, CD34^+^) (*n* = 4, *p < 0.05). (c) Safranin‐O staining of the *Adiscaf* pellets and SVF‐seeded Ultrafoam constructs after chondrogenic differentiation and hypertrophic induction. (d and e) Hypertrophic cartilage constructs engineered from *Adiscaf* pellets and SVF‐seeded Ultrafoam constructs were subcutaneously implanted in nude mice for 8 weeks. H&E staining (d) and microCT scanning (e) of the retrieved implants. (f) Quantification of the GAG concentrations in cell supernatants at the last week of in vitro endochondral induction (*n* = 6, **p < 0.01, compared with the SVF/Ultrafoam constructs). (g) Normalization of the total GAG content to the DNA content in the chondrogenic and hypertrophic constructs (*n* = 6, **p < 0.01, compared with the SVF/Ultrafoam constructs). (h and i) Quantification of the BV/TV (h) and BMD (i) of the *Adiscaf*‐ and SVF/Ultrafoam‐derived implants (*n* = 6; *p < 0.05, **p < 0.01, compared with the SVF/Ultrafoam constructs). (j) Ossicle maturity scoring of the *Adiscaf*‐ and SVF/Ultrafoam‐derived implants (*n* = 6; **p < 0.01, compared with the SVF/Ultrafoam constructs). BMD, bone mineral density; BV/TV, bone volume per tissue volume; EPCs, endothelial progenitor cells; GAG, glycosaminoglycan; H&E, hematoxylin and eosin; SVF, stromal vascular fraction

To compare the potency of *Adiscaf* and SVF‐seeded/Ultrafoam in HyC engineering, ASCs isolated from *Adiscaf* constructs and SVF cells isolated from fresh lipoaspirate were compared in differentiation and phenotype. As shown in Figure 2a, the *Adiscaf* ASCs and fresh SVF cells had comparable differentiation potential toward adipocytes, osteoblasts, and chondrocytes. Furthermore, flow cytometric analysis revealed that the *Adiscaf* constructs had higher percentages of MSCs (CD45^−^ CD73^+^ CD90^+^; *p* < 0.05), pericytes (CD45^−^ CD34^−^ CD146^+^; *p* <0.05), and endothelial progenitor cells (EPCs; CD45^−^ CD31^+^ CD34^+^; *p* < 0.05) than lipoaspirate (Figure 2b). These data indicated that 3 weeks of in vitro proliferating culture not only allowed an abundant expansion of these cells within the adipose tissue but also maintained their differentiation potential. We then compared the efficiency of *Adiscaf* constructs and SVF/Ultrafoam constructs in HyC engineering. Initially, the diameters of *Adiscaf* pellets and Ultrafoam constructs were the same (4 mm), but the number of SVF cells seeded onto the Ultrafoam constructs were significantly greater than the ASCs in the *Adiscaf* pellets. Most interestingly, after 6 weeks of in vitro endochondral priming, although red‐stained GAGs were observed in both *Adiscaf* pellets and SVF/Ultrafoam constructs, the diameter of *Adiscaf* pellets was maintained around 4 mm, and the SVF/Ultrafoam constructs shrunk to approximately 1–2 mm (Figure 2c). The GAG released to supernatants (Figure 2f; *p* < 0.01) and GAG/DNA in constructs (Figure 2g; *p* < 0.01) of the *Adiscaf* pellets were also significantly higher than that in the SVF/Ultrafoam constructs at both chondrogenic differentiation stage and hypertrophic induction stage. The endochondrally primed *Adiscaf* pellets and SVF/Ultrafoam constructs were also subcutaneously implanted into nude mice to test their potency of in vivo bone regeneration. Bone formation in the retrieved constructs was identified by microCT scanning (Figure 2d) analysis. Quantitative analysis showed that the *Adiscaf* pellets had greater bone volume per tissue volume (BV/TV) (Figure 2h; *p* < 0.01) and higher bone mineral density (BMD) (Figure 2i; *p* < 0.05) than SVF/Ultrafoam constructs. H&E staining images further confirmed the presence of bony tissue and bone marrow in both *Adiscaf* pellets and SVF/Ultrafoam constructs (Figure 2e). However, due to the heterogeneous chondrogenic differentiation of the SVF cells within the Ultrafoam, fibrotic tissues also presented in some of the SVF/Ultrafoam constructs. More importantly, the quantified ossicle maturity scores of *Adiscaf* pellets were higher than that of SVF/Ultrafoam constructs (Figure 2j; *p* < 0.01). Collectively, these findings indicate that the in vitro HyC formation and in vivo bone formation potency of the *Adiscaf* constructs is greater than the traditional SVF/Ultrafoam constructs.

### 3.3 Characterization of endochondrally primed *Adiscaf* pellets

**FIGURE 3:**
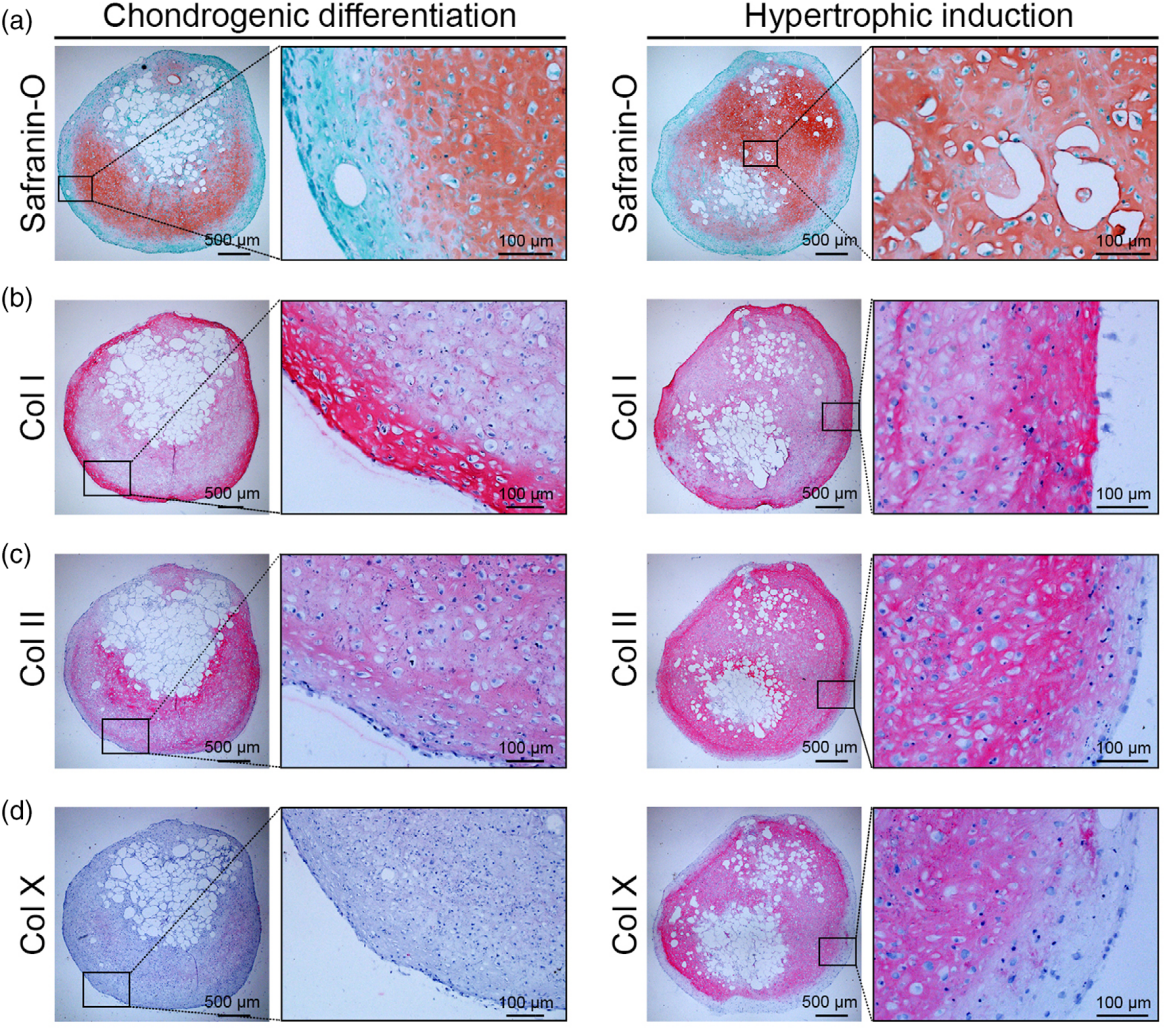
Chondrogenic and hypertrophic priming of the *Adiscaf* pellets. (a) Safranin‐O staining of *Adiscaf* pellets after chondrogenic differentiation and hypertrophic induction. (b–e) IHC staining of Col I (b), Col II (c), and Col X (d) in *Adiscaf* pellets after chondrogenic differentiation and hypertrophic induction. BSP, bone sialoprotein; Col II, collagen type II; Col X, collagen type X; IHC, immunohistochemical; MMP‐9, matrix metalloproteinase‐9; MMP‐13, matrix metalloproteinase‐13

**FIGURE 4:**
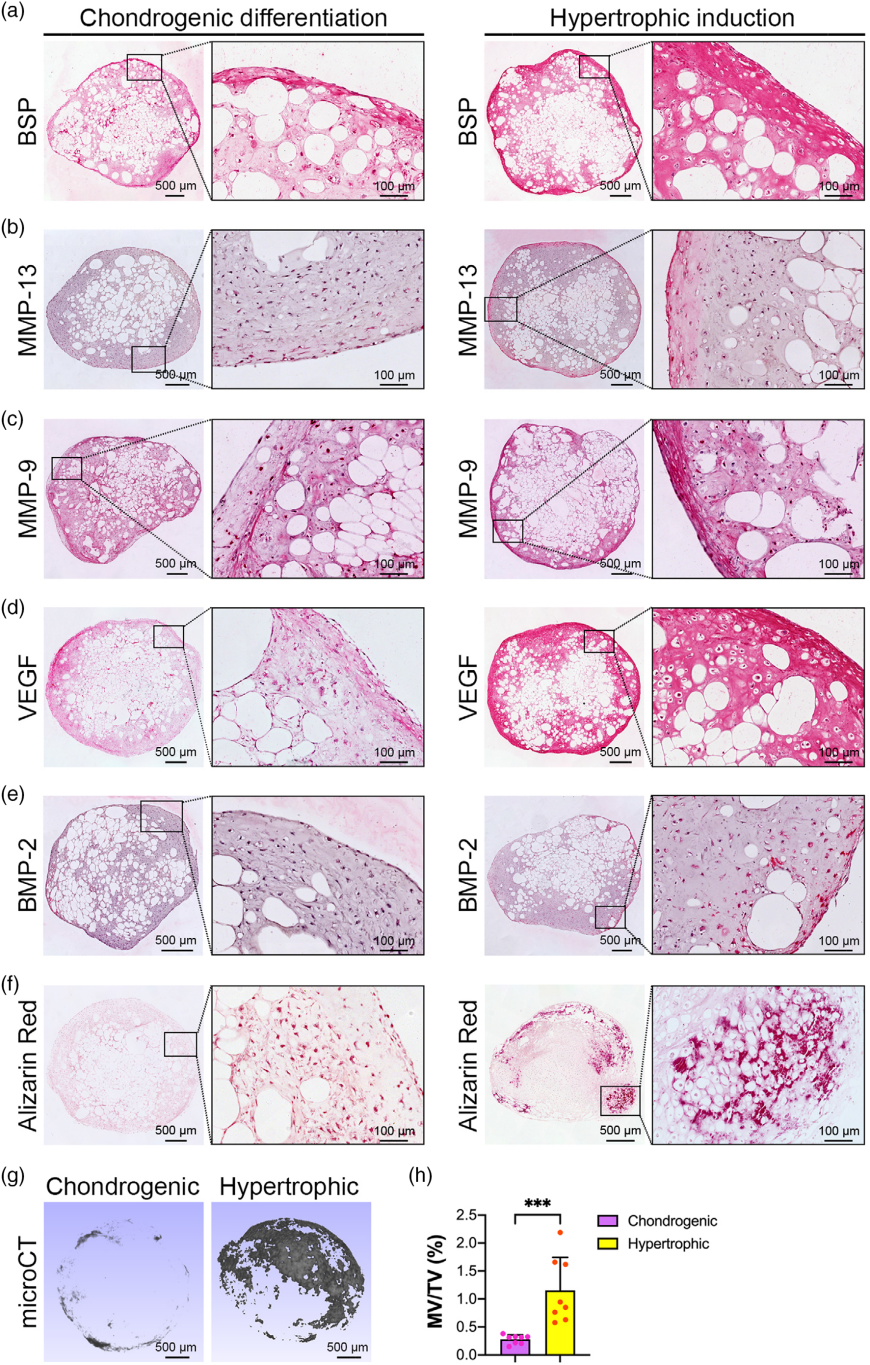
Characterization of endochondrally primed *Adiscaf* pellets. (a and b) IHC staining of BSP (a), MMP‐13 (b), MMP‐9 (c), VEGF (d), and BMP‐2 (e) in *Adiscaf* pellets after chondrogenic differentiation and hypertrophic induction. (f) Alizarin red staining of *Adiscaf* pellets after chondrogenic differentiation and hypertrophic induction, respectively. (g) MicroCT scanning and 3D reconstruction of chondrogenically and hypertrophically primed *Adiscaf* pellets. (h) Quantification of the MV/TV in chondrogenically and hypertrophically primed *Adiscaf* pellets (*n* = 8, ***p < 0.001). BMP‐2, bone morphogenetic protein‐2; IHC, immunohistochemical; MMP‐9, matrix metalloproteinase‐9; MMP‐13, matrix metalloproteinase‐13; MV/TV, mineralized tissue volume per tissue volume; VEGF, vascular endothelial growth factor

The 4 mm *Adiscaf* pellets were chondrogenically primed for 4 weeks, followed by 2 weeks of hypertrophic induction. After 4 weeks of chondrogenic priming, all pellets showed characteristic features of cartilaginous tissue. Histology demonstrated that the pellets displayed abundant deposition of GAGs (Safranin‐O staining, Figure 3a, left), Col I (Figure 3b, left), and Col II deposition (Figure 3C, left). The cells displayed typical round chondrocyte morphology and were embedded in lacunae. A certain degree of hypertrophy was also observed in the chondrogenically primed *Adiscaf* pellets, as evidenced by weak positivity for Col X and BSP (Figure 3d, left and Figure 4a, left). After 2 weeks of hypertrophic induction, the pellets displayed more intense GAG (Figure 3a, right), Col I (Figure 3b, right), and Col II (Figure 3C, right) deposition and were expressed by the hypertrophic markers Col X and BSP (Figure 3d, right and Figure 4a, right), indicating that 6 weeks of endochondral priming converted the *Adiscaf* pellets from adipose tissue into HyC tissue. Cartilaginous matrix remodeling was further confirmed by staining for MMP‐13 and MMP‐9, which are matrix‐degrading enzymes that specifically degrade Col II and proteoglycans in cartilage.^40^, ^41^, ^42^ The chondrogenic‐primed *Adiscaf* pellets initially weakly expressed MMP‐13, and the expression levels were enhanced after hypertrophic induction (Figure 4b). Similarly, MMP‐9 expression was positive in chondrogenically primed *Adiscaf* pellets and significantly enhanced after hypertrophic induction (Figure 4c). These data indicate that *Adiscaf* pellets undergo matrix remodeling during in vitro endochondral priming.

To further analyze the osteogenic and angiogenic potential of the engineered constructs, chondrogenically and hypertrophically primed *Adiscaf* pellets were analyzed for the expression of angiogenesis, osteogenesis, and mineralization mediators, including VEGF, BMP‐2, and calcium deposits. VEGF‐stimulated angiogenesis is critical for ECO, which mediates capillary invasion and osteoclast recruitment, ultimately triggering cartilage remodeling.^43^, ^44^ VEGF expression was observed in the cartilage matrix of chondrogenically primed *Adiscaf* pellets. An additional 2 weeks of hypertrophic induction increased VEGF accumulation (Figure 4d), indicating the angiogenic potential of these pellets. The pellets were initially negative for the expression of the osteogenesis mediator BMP‐2 during chondrogenic priming; however, BMP‐2 expression was later observed in chondrocyte‐like cells, especially in the outer areas of the *Adiscaf* pellets, which were rich in GAGs (Figure 4e). The chondrogenically primed *Adiscaf* pellets were negative for calcium deposits (Alizarin red), but the hypertrophically primed *Adiscaf* pellets displayed a GAG‐rich core surrounded by a mineralized ring (Figure 4f). Mineralized tissue in the *Adiscaf* pellets was further evidenced as a shell around the pellets by microCT (Figure 4g). The percentage of mineralized volume to tissue volume was increased from 0.28% in the chondrogenic differentiation stage to 1.16% in the hypertrophic induction stage (Figure 4h).

### 3.4 Implantation of HyC pellets significantly accelerated critical‐sized calvarial defect healing via ECO

**FIGURE 5:**
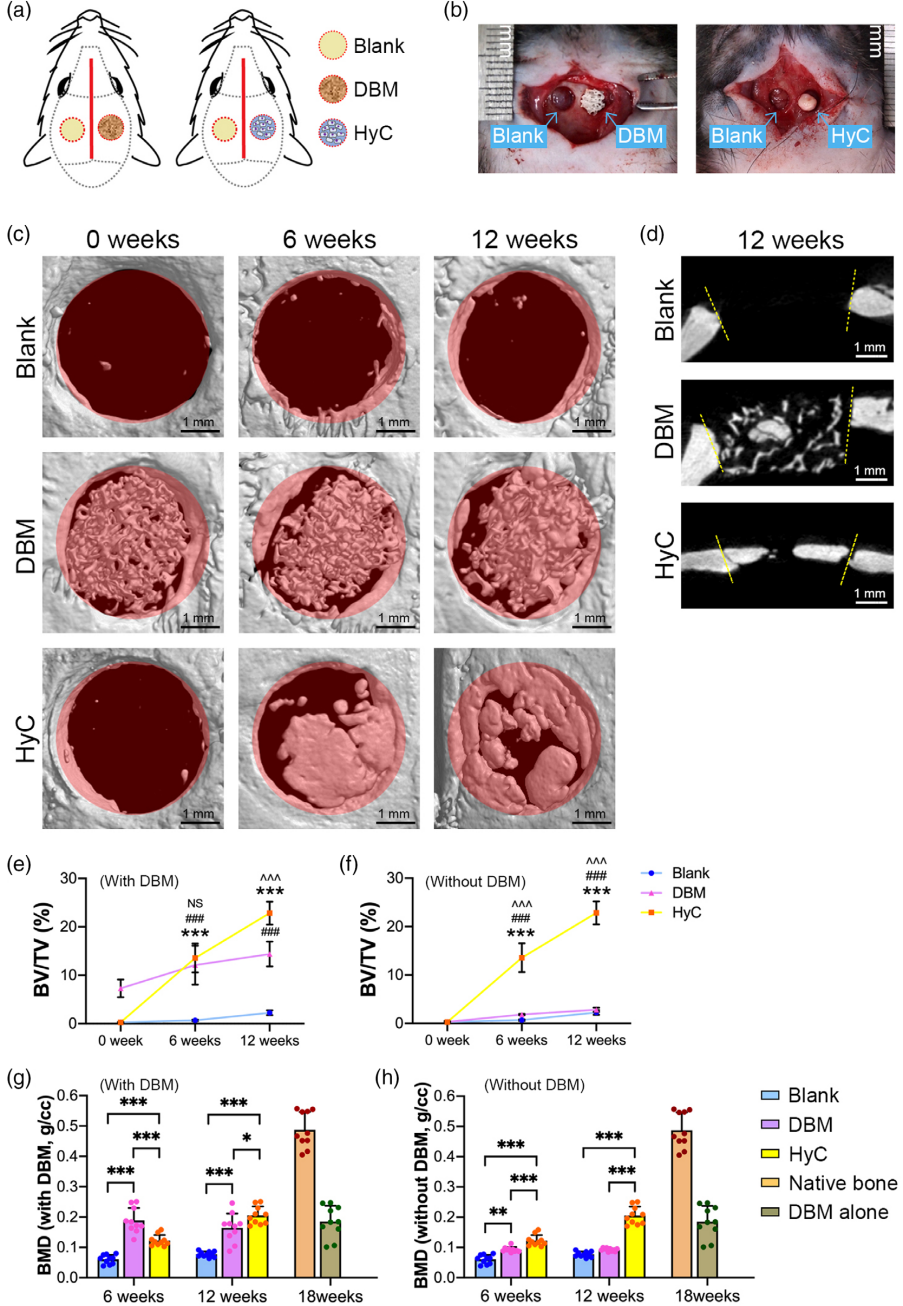
Engineered HyC pellets healed critical‐sized calvarial defects in rats. (a) Schematic diagram of critical‐sized calvarial defect reconstruction using DBM and engineered HyC pellets. (b) Implantation of the DBM graft and HyC pellet into the calvarial defects. (c) 3D reconstructive images of the calvarial defects treated with HyC pellets, DBM grafts, and untreated immediately after the operation and at 6 and 12 weeks after the operation. (d) Cross‐sectional images of the calvarial defects treated with HyC pellet, DBM graft, and left untreated at 12 weeks after the operation. (e and f) Quantification of the BV/TV (with or without the DBM graft) in the calvarial defect area over time (*n* = 10; ***p < 0.001, the HyC group compared with the untreated group; ^###^ *p* < 0.001, the DBM group compared with the untreated group; ^###^ *p* < 0.001, ^^^^^ *p* < 0.001, the DBM group compared with the HyC group). (g and h) Quantification of the BMD (with or without the DBM graft) in the calvarial defect area over time (*n* = 10; *p < 0.05, **p < 0.01, ***p < 0.001). BMD, bone mineral density; BV/TV, bone volume per tissue volume; DBM, decellularized demineralized bone matrix; HyC, hypertrophic cartilage

The in vivo performance of the engineered HyC pellets was compared with that of DBM in an orthotopic immunodeficiency rat calvarial model (Figure 5a,b). MicroCT scans were performed immediately and at 6 and 12 weeks postimplantation to investigate bone regeneration and integration. The 3D reconstructive images showed that in the HyC group, newly formed hard tissue could be observed in the defect space and started to integrate into the adjacent native bone at 6 weeks. By 12 weeks, the newly formed bone tissues covered most of the defect space and had extensive integration along the margin of defects (Figure 5c, bottom). In contrast, in the DBM group, little newly formed hard tissue was present within the trabeculae of the implanted DBM at 6 and 12 weeks, and only minimal integration with the surrounding bone could be observed at 12 weeks (Figure 5c, middle). No obvious hard tissue could be observed within the untreated group at the same time points (Figure 5c, above). The coronal view of the microCT images at 12 weeks showed that the newly formed bone tissue in the HyC group integrated well with the adjacent native bone (Figure 5d). To quantitatively evaluate bone formation in orthotopic sites, bone morphological parameters, including the percentage of BV/TV and BMD, were analyzed. As shown in Figure 5e, when the DBM grafts were calculated as a part of the bone tissue within the defect area, the BV/TV percentages in the HyC and DBM groups were comparable (*p* = 0.27) and significantly higher (*p* < 0.001) than those in the untreated group at 6 weeks. After 12 weeks of orthotopic implantation, the BV/TV percentage in the HyC group was markedly increased, approximately 1.6 times higher than that in the DBM group. Meanwhile, the BMD of the HyC group was initially lower (*p* < 0.001) than that of the DBM group at 6 weeks but gradually increased and higher than that of the DBM group at 12 weeks (Figure 5g), which was approximately half that of the native calvarial bone (Figure S1c), indicating that the newly formed bone tissue underwent remodeling within the defects. To quantitatively evaluate the newly formed bone tissue, the volume and BMD of the DBM grafts themselves were excluded from the bone tissue within the defects based on microCT data obtained from DBM alone (Figure S1a,b). The results revealed that the BV/TV percentage of new bone in the HyC group was over seven‐fold higher than that in the DBM group at 6 weeks and over eight‐fold higher at 12 weeks. The BV/TV percentage of new bone in the DBM group was slightly higher than that in the untreated group at both 6 and 12 weeks (Figure 5f). The BMD levels of new bone in all three groups demonstrated the same tendencies as the BV/TV percentages (Figure 5h).

**FIGURE 6:**
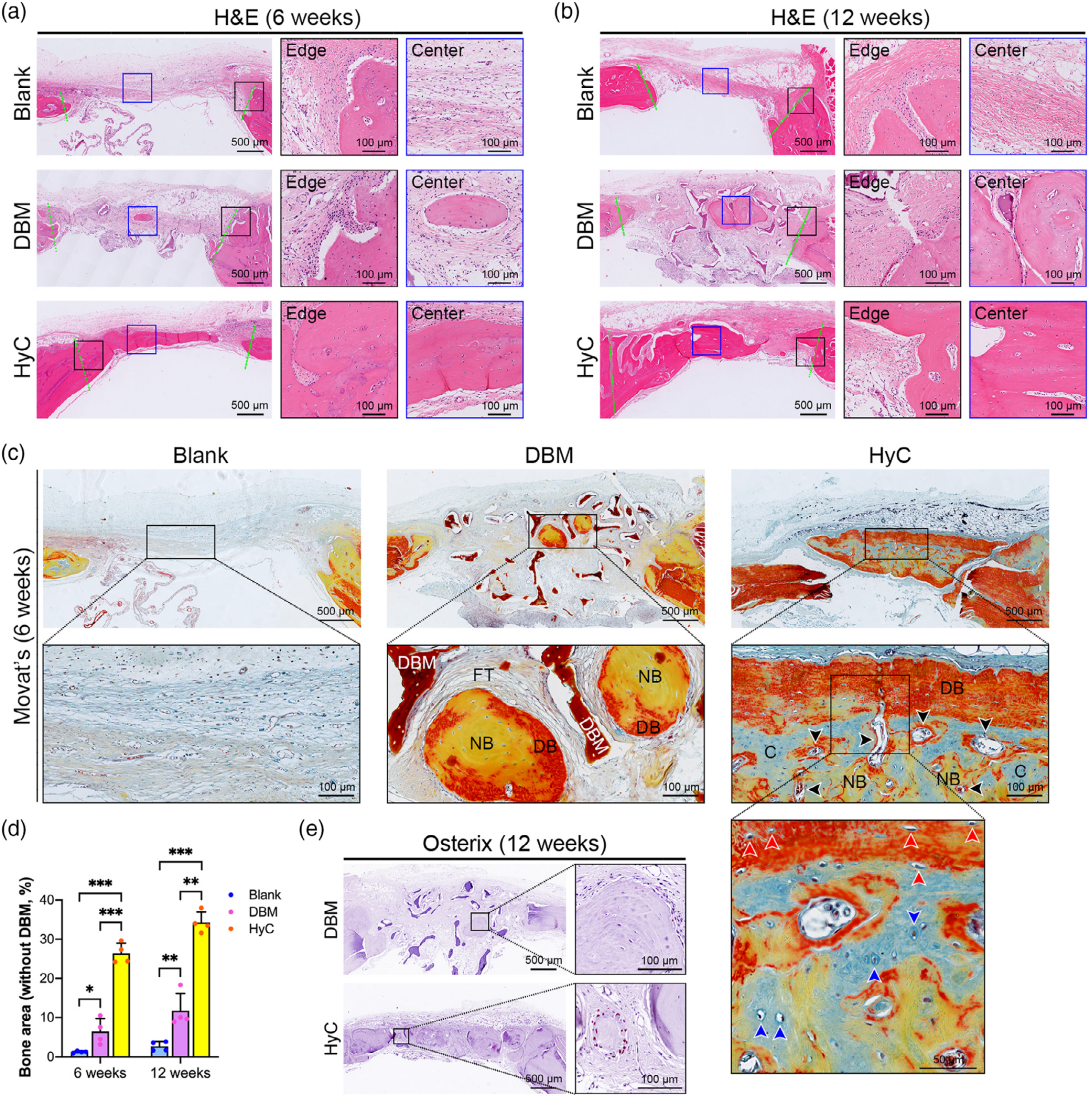
Engineered HyC pellets supported new bone formation by recapitulating ECO. (a and b) H&E staining of calvarial defects from each group at 6 (a) and 12 (b) weeks postimplantation. (c) Movat's pentachrome staining of the calvarial defects at 6 weeks postimplantation (black arrows indicate blood vessels, blue arrows indicate hypertrophic chondrocytes, red arrows indicate osteoblasts/osteocytes). (d) Histomorphometric analysis of the healing area (without DBM) of each group (*n* = 4, *p < 0.05, **p < 0.01, ***p < 0.001). (e) IHC staining of Osterix in the DBM and HyC‐implanted defects at 12 weeks postimplantation. C, cartilage analog; DB, dense bone; DBM, decellularized demineralized bone matrix; ECO, endochondral ossification; FT, fibrous tissue; H&E, hematoxylin & eosin; HyC, hypertrophic cartilage; IHC, immunohistochemical; NB, new bone

New bone formation was further confirmed by histological analysis. Bone tissue formation in the defect space was identified by H&E (Figure 6a,b) and Masson (Figure S2a,b) staining in both the DBM and HyC groups. The new bone tissue demonstrated characteristic osteocytes embedded inside the bone matrix and cuboidal osteoblasts at the periphery of the bone matrix that were positive for the osteoblastic marker Osterix (Figure 6e). In the untreated group, most of the defect was filled by thin fibrous tissue lacking bone tissue at both 6 and 12 weeks postimplantation (Figure 6a,b, above). In the DBM group, the DBM grafts were visible and deep red in color, and the DBM grafts were still acellular. A small amount of new bone tissue, which showed a light red color with cells present, could be identified among the trabeculae of the DBM at both 6 and 12 weeks postoperation (Figure 6a,b, middle). However, no evidence of new bone formation was observed by H&E and Masson staining when the DBM grafts were implanted subcutaneously at ectopic sites in nude mice (Figure S3a,b). In the HyC group, a substantial level of dense bone tissue was observed in the defect space and integrated well with adjacent native bone at 6 weeks, which had repaired almost the entire calvarial defect at 12 weeks (Figure 6a,b, bottom). The newly formed bone tissue became thicker and more extensively remodeled, resulting in interconnected trabecular‐like bone formation 12 weeks after implantation (Figure 6b, bottom). Quantitative analysis revealed that the percentages of new bone increased in the DBM and HyC groups over time since implantation increased. The percentages of the new bone area in the HyC group were three to four times larger than those in the DBM group (Figure 6d). Movat's pentachrome staining, by which the cartilage matrix was stained blue, the dense bone tissue was stained red, and the newly formed bone tissue was stained yellow, was performed to investigate the histological characteristics of the newly formed bone tissue within the defects (Figure 6c). In the DBM group, the DBM grafts were stained deep red, and no cells were observed within the grafts. The core of the newly formed bone tissue was stained yellow, and the outer area was stained red, but no blue staining was found in the defect area, indicating that the new bone tissue may have formed via IMO (Figure 6c, middle). In the HyC group, the outer area of the newly formed bone tissue with cuboidal osteoblasts/osteocytes (red arrows) was stained red, and the core consisted of yellow‐stained trabecular‐like bone matrix and blue‐stained cartilage analogs. More importantly, blood vessels (black arrows) and enlarged hypertrophic chondrocytes (blue arrows) were observed in the cartilage analogs, indicating that bone tissue was formed via ECO in the HyC group (Figure 6c, right).

### 3.5 The newly formed bone tissue originated from human adipose tissue and underwent remodeling in vivo

**FIGURE 7:**
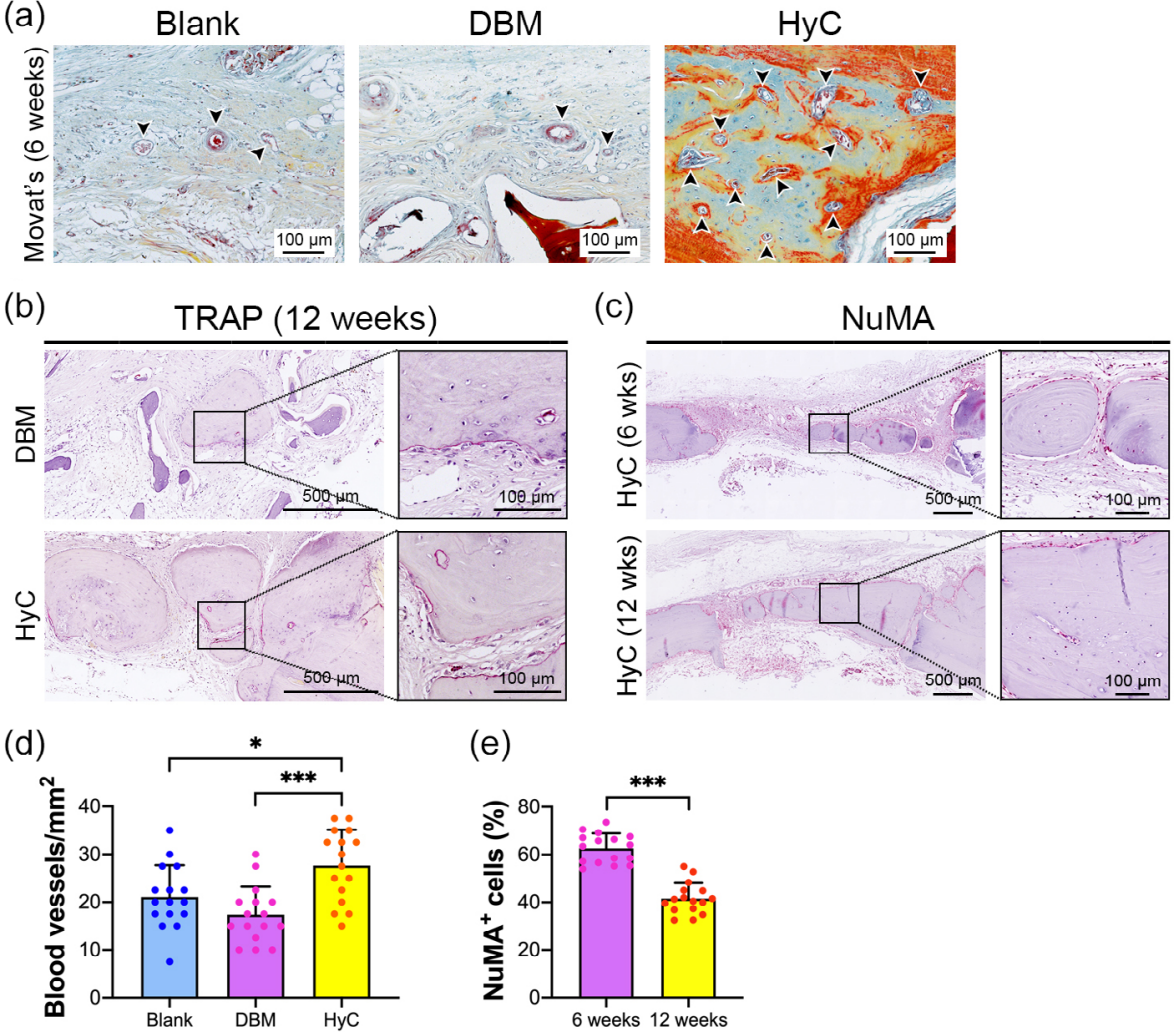
The newly formed bone tissue originated from human tissue and underwent remodeling. (a) Movat's pentachrome staining of the calvarial defect from the three groups (black arrows show blood vessels). (b) TRAP staining of the defects implanted with DBM and HyC constructs 12 weeks postimplantation. (c) IHC staining of NuMA in HyC‐implanted defects at 6 and 12 weeks postimplantation. (d) Quantitative analysis of blood vessel invasion within the defects from the HyC, DBM, and untreated groups at 6 weeks postimplantation (*n* = 16, *p < 0.05, ***p < 0.001). (e) Quantification of NuMA‐positive cells in the HyC‐implanted defects at 6 and 12 weeks postimplantation (*n* = 16, ***p < 0.001). DBM, decellularized demineralized bone matrix; HyC, hypertrophic cartilage; IHC, immunohistochemical; NuMA, nuclear mitotic apparatus protein; TRAP, tartrate‐resistant acid phosphatase

Hypertrophic chondrocytes have the intrinsic ability to secrete angiogenic factors, which promote capillary invasion and regulate bone regeneration and remodeling during development.^25^, ^45^ As shown in Figure 7a, Movat's staining images demonstrated that the blood vessels mainly existed in the fibrous tissue and barely appeared in newly formed bone tissue in the untreated and DBM groups at 6 weeks after implantation. However, in the HyC group, abundant blood vessels were scattered in the defect area, especially in newly formed bone tissue. The blood vessel density of the defect area was denser in the HyC group than in the DBM and untreated groups (Figure 7d). Intensive bone resorption by osteoclasts, characterized as TRAP‐positive multinucleated cells, was observed in the margins of newly formed bone tissue in both the DBM and HyC groups (Figure 7b), suggesting that the newly formed bone tissues underwent extensive remodeling in the calvarial defects. To investigate the origin of newly formed bone tissue, the samples were stained with a human NuMA antibody, which does not cross‐react with rat tissue. NuMA‐positive cells were present throughout the newly formed bone tissue and their surrounding tissue in the HyC‐implanted defects at both 6 and 12 weeks (Figure 7c). In contrast, NuMA‐positive cells were not detected in the untreated and DBM‐implanted defects, even in the newly formed bone tissue of the DBM group (Figure S4a,b). These data indicate that the new bone within the DBM‐implanted defects might be formed via the recruitment of host cells, whereas the new bone within the HyC‐implanted defects originated from human adipose tissues. Interestingly, the number of NuMA‐positive cells in the new bone tissue tended to decrease from 6 to 12 weeks postimplantation (Figure 7e), suggesting that human cells might be gradually replaced by host cells over time.

## 4 DISCUSSION

Cell‐based EBR approaches have been previously approved as a potential strategy for large bone defect reconstruction. However, recent efforts have proven insufficient for clinical translation due to limitations, such as complex engineering procedures, poor in vitro differentiation efficiencies, and incomplete bone regeneration at orthotopic sites.^32^, ^33^, ^34^ In this study, we developed a novel adipose tissue‐based developmental engineering approach for the biofabrication of HyC intermediate tissues. These engineered HyC constructs were sufficient to heal critical‐sized calvarial defects via ECO in a rat model. The pellets resulted in increased bone formation, a higher blood vessel density, and better integration of the implants with the adjacent native bone when compared with DBM implants and empty defects. This effect may be due to the remarkable capacity of the HyC pellets to secrete angiogenic and osteogenic signals, including VEGF, BMP‐2, and calcium nodules. The approach demonstrated in this study successfully tested the feasibility of repairing large bone defects using adipose tissue engineered HyC constructs and offers an alternative therapeutic option to bone defect repair in the clinic.

A prerequisite for harnessing developmental engineering strategies for large bone defect repair is engineering a HyC intermediate in vitro, which subsequently stimulates EBR in orthotopic sites. An ideal cell source for endochondral bone engineering must have the capacity to undergo hypertrophic chondrocyte differentiation and synthesize HyC‐specific ECM. ASCs are a heterogeneous population of cells in subcutaneous adipose tissue that exhibit several distinct advantages over cells from other sources.^46^, ^47^ BMSCs and PDCs have shown higher chondrogenic potential differentiation and greater Col X expression capacity than ASCs.^48^ ASCs are easily accessible in abundant quantities by a minimally invasive procedure and have a higher proliferative capacity.^11^, ^49^ More importantly, ASCs, either cultured as micromass pellets^50^, ^51^ or spheroids^45^ or seeded onto collagen sponges,^36^, ^50^ can mature into HyC tissues under in vitro chondrogenic priming conditions and recapitulate ECO in vivo, suggesting their clinical translation potential for bone defect reconstruction. However, the clinical translation of these ASC‐based approaches is still limited by complex cell isolation, expansion, and loading procedures. Furthermore, ASCs easily lose their primitive stemness and exhibit reduced proliferation potential and cell senescence during long‐term in vitro expansion.^52^, ^53^, ^54^ Evidence that a native ECM microenvironment actively preserves the stemness and differentiation potential of mesenchymal progenitor cells was confirmed in different stem cells,^55^, ^56^ including BMSCs,^57^ ASCs,^34^ and periodontal ligament cells.^58^ In this study, we directly cultured fractioned adipose tissue in a proliferative medium, where the cells expanded well within their own adipose ECM. Our data showed that the *Adiscaf* constructs possess greater in vitro HyC formation and in vivo bone formation potency than the traditional approach SVF/Ultrafoam constructs. The distribution of proliferating ASCs and chondrocytes are heterogeneous in the *Adiscaf* constructs. Proliferating cells and subsequent differentiated chondrocytes are frequently located in the outer region of the constructs due to heterogeneous medium diffusion in the inner region. Therefore, an additional week of proliferative culture of *Adiscaf* constructs in the form of 4‐mm pellets was performed to increase cell density and homogeneity in this study. After sequential chondrogenic differentiation and hypertrophic induction, the *Adiscaf* pellets expressed increased levels of HyC markers, including Col II, Col X, and BSP, indicating the successful conversion of adipose tissue into HyC tissue. In addition, the upregulated expression of MMP‐13 and MMP‐9 in the engineered HyC pellets further confirmed the remodeling of cartilaginous ECM between the early and late hypertrophic stages after endochondral priming.

Implantation of HyC intermediates instead of bone tissue‐like constructs into defects to promote bone formation is an essential difference between developmental engineering strategies and traditional IMO‐based strategies. HyC intermediate implantation approaches are driven by the fact that hypertrophic chondrocytes can secrete angiogenic and osteogenic factors that play pivotal roles in both the vascularization of constructs in vivo and the deposition of a mineralized matrix, resulting in bone deposition.^59^ In this study, we confirmed that hypertrophic induction induced abundant VEGF and BMP‐2 accumulation and mineral deposition in *Adiscaf* pellets, which was consistent with the results of previous studies in which the expression of BMPs and VEGF at the mRNA or protein level was also upregulated in HyC constructs engineered from ASCs or BMSCs.^26^, ^45^, ^50^ The capacity of HyC pellets to express VEGF and BMP‐2 and exhibit mineral deposition may be responsible for the greater percentage of bone volume and higher blood vessel density obtained with these pellets than with the traditional DBM grafts in this study.

We previously reported that HyC constructs engineered from human adipose tissue can remodel into bone organs containing abundant bone matrix and marrow components upon ectopic implantation, indicating their potential for bone defect repair.^36^, ^37^ In the present study, we further investigated the capacity of adipose‐derived HyC constructs to enhance healing by recapitulating ECO in a critical‐sized calvarial defect model. To evaluate the bone regeneration capacity of the HyC pellets, DBM grafts, which were manufactured from pig femur cancellous bone by decellularization and demineralization, were used as a control to mimic conventional clinical treatment for bone defects. New bone formation was observed in both the HyC and DBM groups at both 6 and 12 weeks after implantation; no new bone formation was observed in empty defects. The DBM grafts were shown to be unable to form new bone tissue but provoked a certain degree of bone absorption at ectopic sites in nude mice, as evidenced by the presence of osteoclasts on the surface of DBM grafts (Figure S3a). The newly formed bone within the trabeculae of DBM grafts in orthotopic sites may be induced by their osteoinductive and osteoconductive capacities, which recruit host stem cells into the defect space and induce bone formation under the defect microenvironment. New bone formation within the HyC‐implanted defects occurred via the ECO pathway, as evidenced by the presence of cartilage analog within the core region of the new bone and the expression of human NuMA by some chondrocytes and osteoblasts/osteocytes within the new bone. Most importantly, the HyC pellets demonstrated regenerative superiority over DBM grafts, yielding an increased percentage of new bone volume, a higher BMD, higher blood vessel density, and better integration with the adjacent native bone at both time points. In clinical scenarios, the implanted DBM is usually considered a part of the hard tissue in repaired defects. Even taking this factor into account, the percentage of total BV in the DBM group was comparable to that in the HyC group at 6 weeks but significantly lower at 12 weeks, which may result from the greater new bone formation in the HyC group and possible DBM absorption. In this study, we employed a critical‐sized calvarial bone defect model, where was considered to provide an IMO‐inducing healing microenvironment, to investigate the bone forming capacity of the adipose tissue‐derived HyC pellets. We observed the coexistence of both bone and HyC in the implanted HyC pellets, confirming that the bone formation in the calvarial defect was mainly driven by the HyC pellets themselves. The superior bone regeneration induced by the HyC pellets was likely due to the progression of natural ECO, as was shown for long bone^24^, ^30^ or calvarial bone^60^ defect repair using chondrocyte pellets implanted into the defects. However, the calvarial bone defect models natively lack mechanical stimuli, which have been proven to promote EBR and neovascular invasion.^61^

A major obstacle to clinical translation of this approach is the long‐term in vitro endochondral priming period required for preparing HyC pellets before application in the current engineering protocol. Furthermore, due to immunogenicity and individual variation in the differentiation potential of donor adipose tissue, HyC constructs engineered from human adipose tissue can only be used as autografts, and they mediate bone regeneration in a donor‐dependent manner, lending additional uncertainty to clinical translation. One solution for overcoming these issues is developing a decellularized HyC matrix from living HyC pellets as an off‐the‐shelf and immunecompatible alternative. Recently, decellularized HyC matrices, developed by chemical, enzymatic, and physical procedures^62^, ^63^, ^64^, ^65^ from different living HyC tissues, have been shown to have the capacity to directly attract endogenous MSCs toward the scaffold by leveraging bioactive cues embedded within the decellularized HyC matrix.^66^ These matrices can also be activated by living cells before implantation^67^, ^68^ to initiate the ECO process and EBR.

## 5 CONCLUSION

Here, we are the first to demonstrate the feasibility of repairing large bone defects with engineered HyC constructs generated from human subcutaneous adipose tissue, which provide the necessary angiogenic and osteogenic signals following implantation to enhance vascularization, bone formation, and remodeling in vivo. The results demonstrate that the engineered HyC constructs can support bone formation in orthotopic defects by recapitulating the ECO process and showed higher efficiency in promoting bone regeneration, integration, and vascularization than traditional DBM grafts and untreated defects. This study demonstrates a new therapeutic option for repairing large bone defects with human adipose‐derived HyC grafts in clinical conditions.

## AUTHOR CONTRIBUTIONS

**Ru‐Lin Huang:** Conceptualization (equal); data curation (equal); formal analysis (equal); funding acquisition (equal); investigation (equal); methodology (equal); project administration (equal); resources (equal); supervision (equal); visualization (equal); writing – original draft (equal); writing – review and editing (equal). **Rao Fu:** Data curation (equal); formal analysis (equal); investigation (equal); methodology (equal); software (equal); writing – original draft (equal). **Yuxin Yan:** Data curation (equal); methodology (equal); writing – original draft (equal). **Chuanqi Liu:** Data curation (equal); investigation (equal); methodology (equal). **Jing Yang:** Data curation (equal); investigation (equal); methodology (equal).

## CONFLICTS OF INTEREST

The authors declare no conflicts of interest.

### PEER REVIEW

The peer review history for this article is available at https://publons.com/publon/10.1002/btm2.10312.

## Supporting information


**FIGURE S1** (a) 3D reconstructive images of the DBM graft and native calvarial bone. (b) Cross‐sectional images of the DBM graft and native calvarial bone. (c) Quantification of the BMD in the DBM graft and native calvarial bone (n = 10; *** *P* <0.001). Abbreviation: DBM, decellularized demineralized bone matrix.

**FIGURE S2** Masson trichrome staining of the calvarial defects from the 3 groups at 6 (a) and 12 weeks (b) postimplantation. Abbreviations: HyC, hypertrophic cartilage; DBM, decellularized demineralized bone matrix.

**FIGURE S3** H&E (a) and Masson (b) staining of the DBM constructs after 8 weeks of subcutaneous implantation in nude mice (black arrows show osteoclasts). Abbreviations: HE, hematoxylin & eosin; DBM, decellularized bone matrix.

**FIGURE S4** IHC staining of NuMA in the untreated defects (a) and the DBM‐treated defects (b) at 12 weeks postimplantation. Abbreviations: DBM, decellularized demineralized bone matrix; NuMA, nuclear mitotic apparatus protein.

## Data Availability

The data that supports the findings of this study are available in the supplementary material of this article.
