# Supplementary figures and images for: Engineering hypertrophic cartilage grafts from lipoaspirate for critical‐sized calvarial bone defect reconstruction: An adipose tissue‐based developmental engineering approach
Source: Bioeng Transl Med. 2022 Mar 24;7(3):e10312. doi: 10.1002/btm2.10312 (PMC9472001; doi:10.1002/btm2.10312)

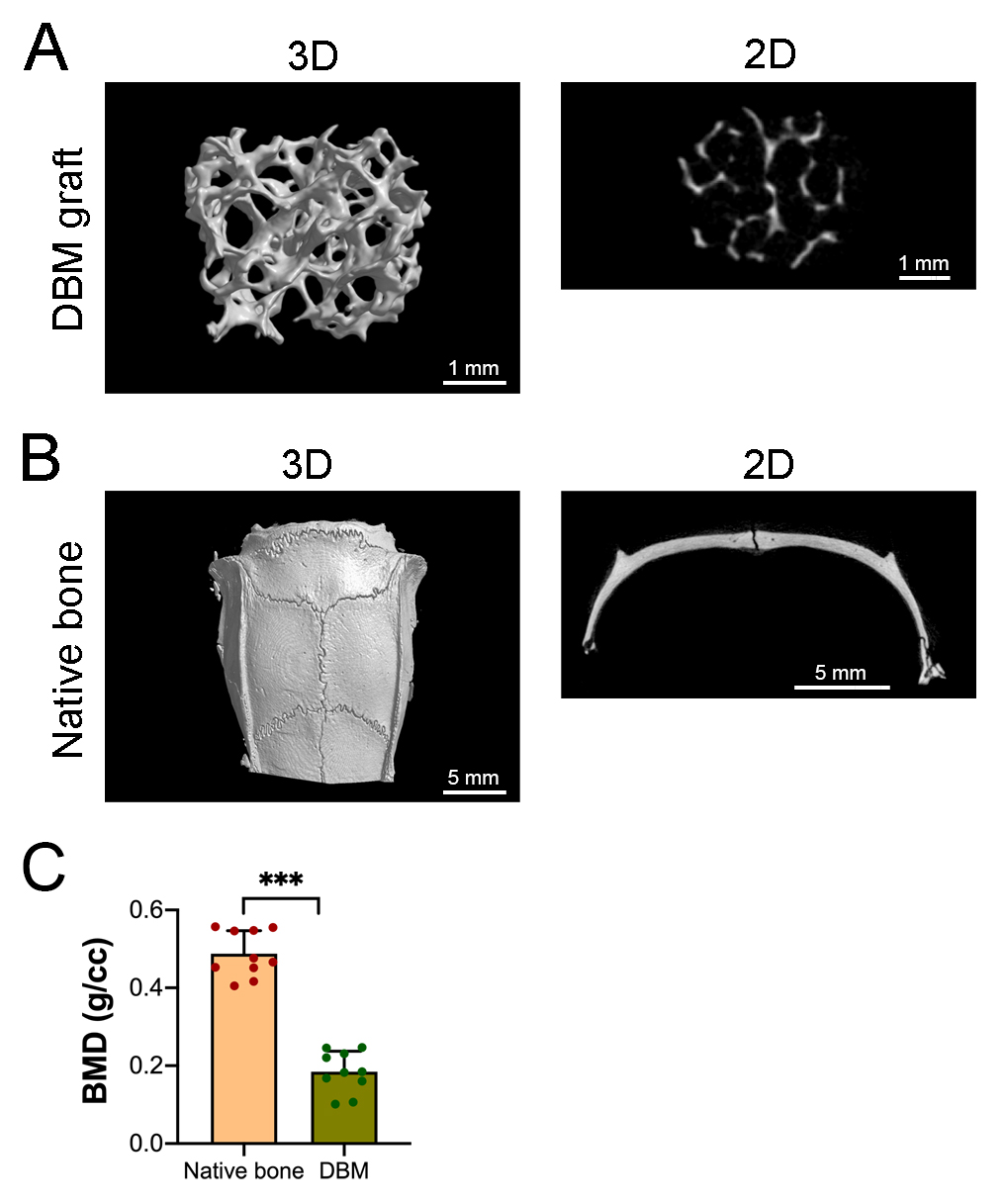

Supplement: Supplementary file 1 — FIGURE S1 (a) 3D reconstructive images of the DBM graft and native calvarial bone. (b) Cross‐sectional images of the DBM graft and native calvarial bone. (c) Quantification of the BMD in the DBM graft and native calvarial bone (n = 10; *** P <0.001). Abbreviation: DBM, decellularized demineralized bone matrix. [file BTM2-7-e10312-s004.jpg]

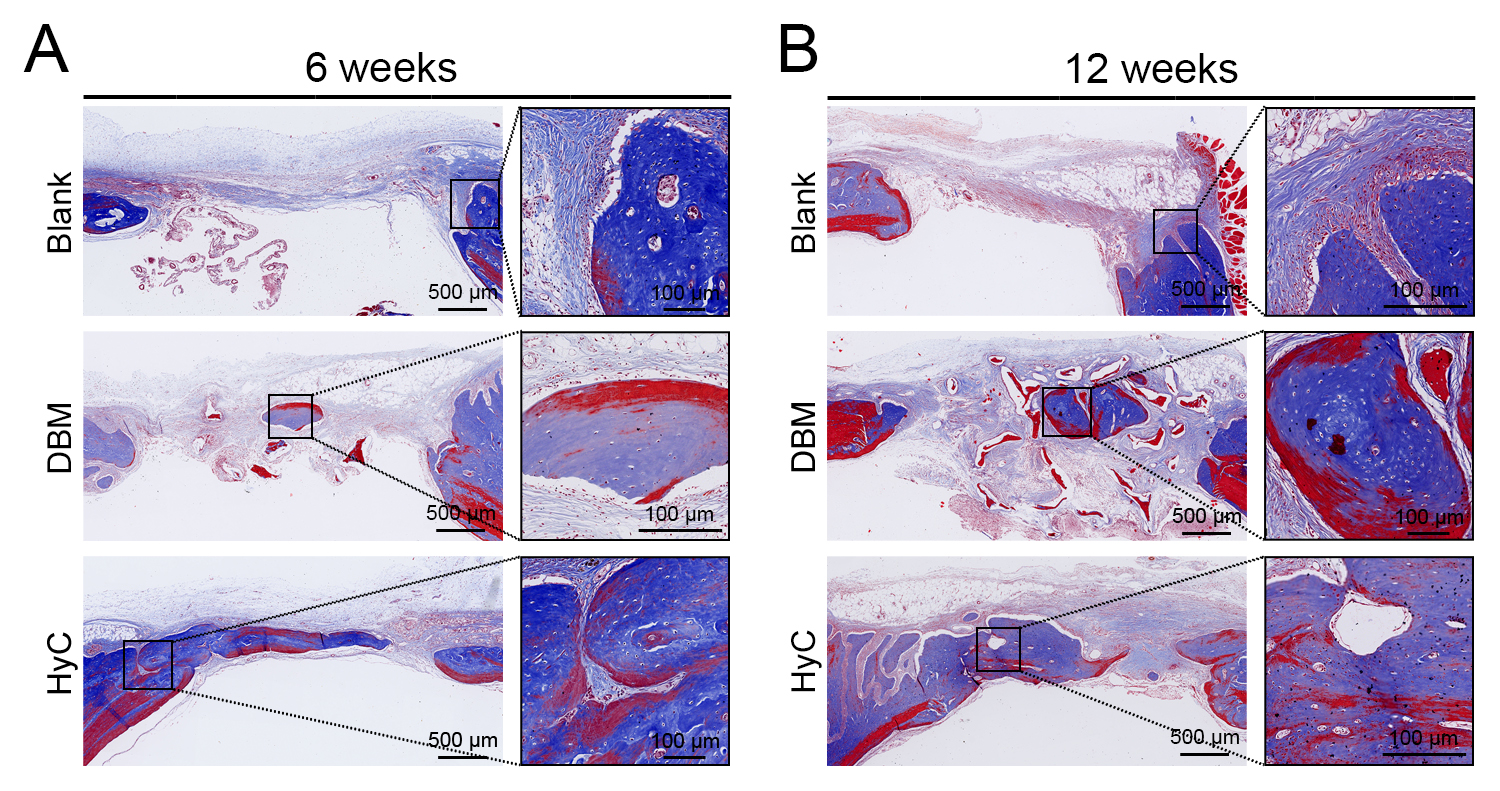

Supplement: Supplementary file 2 — FIGURE S2 Masson trichrome staining of the calvarial defects from the 3 groups at 6 (a) and 12 weeks (b) postimplantation. Abbreviations: HyC, hypertrophic cartilage; DBM, decellularized demineralized bone matrix. [file BTM2-7-e10312-s003.jpg]

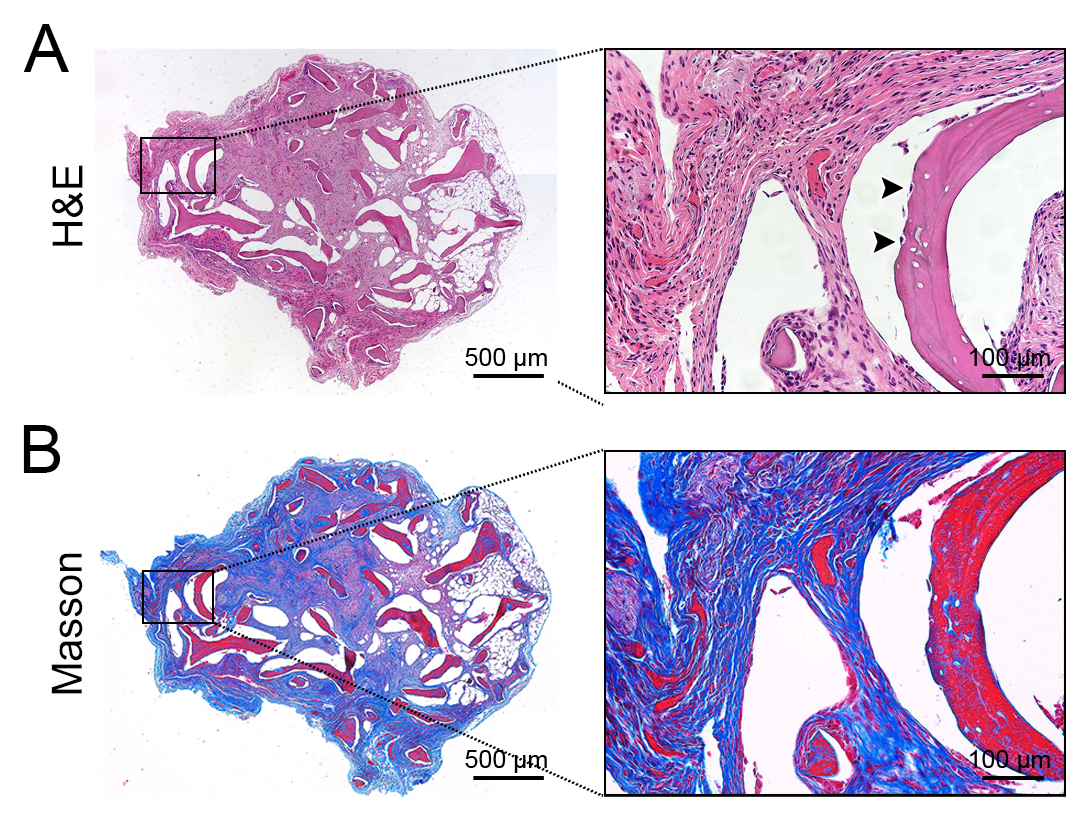

Supplement: Supplementary file 3 — FIGURE S3 H&E (a) and Masson (b) staining of the DBM constructs after 8 weeks of subcutaneous implantation in nude mice (black arrows show osteoclasts). Abbreviations: HE, hematoxylin & eosin; DBM, decellularized bone matrix. [file BTM2-7-e10312-s002.jpg]

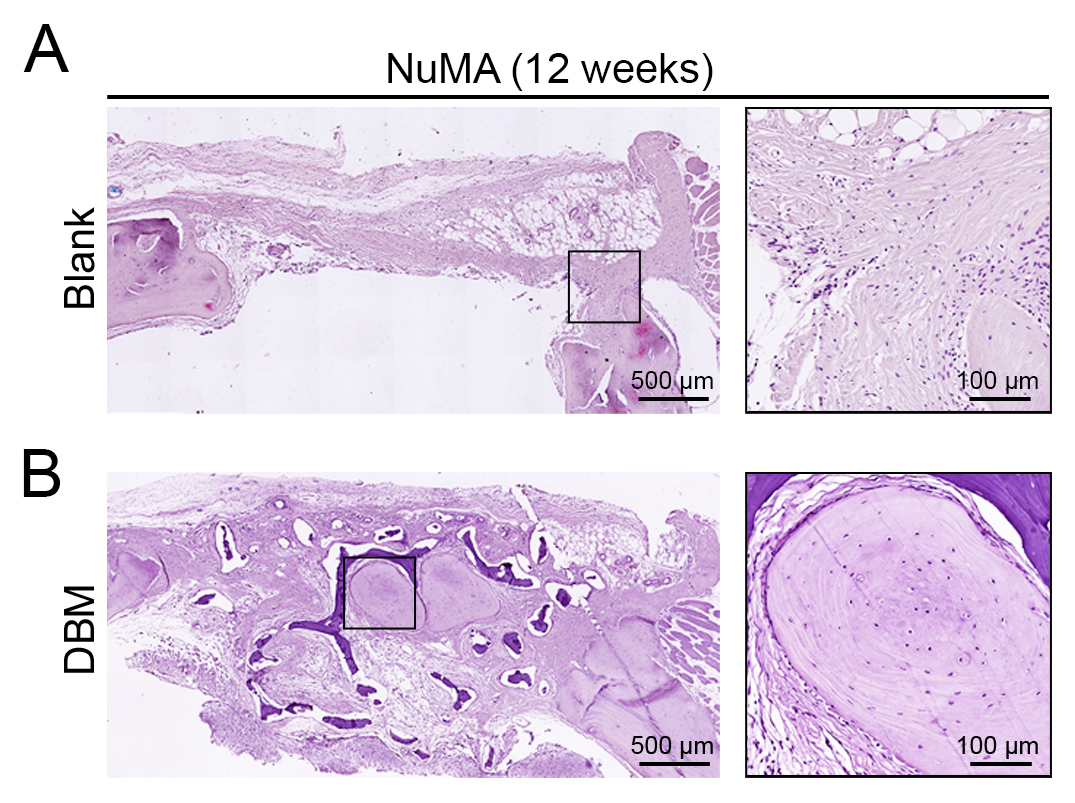

Supplement: Supplementary file 4 — FIGURE S4 IHC staining of NuMA in the untreated defects (a) and the DBM‐treated defects (b) at 12 weeks postimplantation. Abbreviations: DBM, decellularized demineralized bone matrix; NuMA, nuclear mitotic apparatus protein. [file BTM2-7-e10312-s001.jpg]
